# Price, availability and affordability of medicines

**DOI:** 10.4102/phcfm.v6i1.604

**Published:** 2014-06-24

**Authors:** Brenda S. Mhlanga, Fatima Suleman

**Affiliations:** 1 Discipline of Pharmaceutical Sciences, School of Health Sciences, University of KwaZulu-Natal, South Africa

## Abstract

**Background:**

Medicines play an important role in healthcare, but prices can be a barrier to patient care. Few studies have looked at the prices of essential medicines in low- and middle-income countries in terms of patient affordability.

**Aim:**

To determine the prices, availability and affordability of medicines along the supply chain in Swaziland.

**Setting:**

Private- and public-sector facilities in Manzini, Swaziland.

**Methods:**

The standardised methodology designed by the World Health Organization and Health Action International was used to survey 16 chronic disease medicines. Data were collected in one administrative area in 10 private retail pharmacies and 10 public health facilities. Originator brand (OB) and lowest-priced generic equivalent (LPG) medicines were monitored and these prices were then compared with international reference prices (IRPs). Affordability was calculated in terms of the daily wage of the lowest-paid unskilled government worker.

**Results:**

Mean availability was 68% in the public sector. Private sector OB medicines were priced 32.4 times higher than IRPs, whilst LPGs were 7.32 times higher. OBs cost 473% more than LPGs. The total cumulative mark-ups for individual medicines range from 190.99% – 440.27%. The largest contributor to add-on cost was the retail mark-up (31% – 53%). Standard treatment with originator brands cost more than a day's wage.

**Conclusion:**

Various policy measures such as introducing price capping at all levels of the medicine supply chain, may increase the availability, whilst at the same time reducing the prices of essential medicines for the low income population.

## Abstrait

**Le prix, la disponibilité et l'accessibilité financière des médicaments**.

**Contexte:**

Les médicaments jouent un rôle important dans les soins médicaux, mais les prix peuvent être un obstacle aux soins prodigués au patient. Peu d’études se sont penchées sur le prix des médicaments essentiels dans les pays à revenu faible et intermédiaire pour ce qui est des prix abordables pour le patient.

**Objectif:**

Déterminer les prix, la disponibilité et l'accessibilité financière des médicaments sur le réseau de distribution au Swaziland.

**Location:**

Les établissements privés et du secteur public à Manzini, au Swaziland.

**Méthodes:**

On a utilisé la méthodologie standardisée conçue par l'Organisation Mondiale de la Santé et l'Action Internationale de la Santé pour étudier 16 médicaments pour maladies chroniques. Les données ont été collectées dans une région administrative, dans 10 pharmacies de détail privées et dans 10 établissements de santé publique. Les médicaments de marque originale (OB) et les équivalents génériques les moins chers (LPG) ont été contrôlés et ces prix ont été comparés aux prix internationaux de référence (IRP). Les prix abordables ont été calculés en fonction du salaire minimum journalier d'un fonctionnaire peu qualifié.

**Résultats:**

La disponibilité était de 68% dans le secteur public. Les médicaments de marque originale (OB) du secteur privé coûtaient 32.4 fois plus cher que les IRP, alors que les LPG coûtaient 7.32 fois plus cher. Les OB coûtaient 473% plus cher que les LPG. Les majorations cumulatives totales des médicaments individuels vont de 190.99% à 440.27%. Le facteur majeur du coût additionnel était la majoration de détail (31% – 53%). Le traitement normal avec un médicament de marque originale coûte plus qu'une journée de salaire.

**Conclusion:**

Différentes mesures telles que l'introduction d'un plafonnement des prix à tous les niveaux de la chaîne de ravitaillement des médicaments pourraient augmenter la disponibilité, tout en réduisant le prix des médicaments essentiels pour la population à faibles revenus.

## Introduction

Access to medicines is influenced by many factors such as affordability, rational use, sustainable financing and reliable supply systems. One of the elements restricting access to medicines is high medicine prices.^1^ This can have a detrimental effect on patients’ health as well as the healthcare system in terms of lack of patient compliance with treatment and subsequent hospitalisation for serious complications. To increase access to medicines, one would thus need to ensure that medicines are affordable in order to counteract any existing barriers that might hinder medicine access. The stated goal of the 2011 Swaziland National Pharmaceutical Policy (SNPP) was to:

…[*c*]ontribute to improving the health of the Swaziland population by ensuring equitable access to and rational use of efficacious, good quality essential medicines and medical supplies and devices at affordable cost, particularly for vulnerable populations.^2^

One way of measuring affordability is to adjust prices by average income per person. This aids in understanding the value of a medicine with respect to the amount of income the average person has available to spend on pharmaceuticals. The SNPP has as objectives the development of pricing policies for medicines and encouraging generic prescribing and dispensing as well as generic substitution. These are meant to be part of future regulatory interventions.

Swaziland has a gross domestic product (GDP) per capita income of USD3073 and is classified as a lower middle-income country.^3^ About 63% of the population are living below the upper poverty line of USD8.21 per capita per month.^3^ The country's revenue has been affected severely by the global downturn. Southern African Customs Union (SACU) receipts were approximately 60% of government revenue in 2008. They were estimated at 9.3% in 2010. The 2006–2007 Demographic Health Survey (DHS) indicated that Swaziland was off track with regard to meeting its Millennium Development Goal (MDG) with health outcomes worsening as a result of high levels of HIV, AIDS and tuberculosis and limited progress with maternal, neo-natal and child health.^4^

The government, private sector, non-governmental organisations (NGOs) and faith-based organisations are all involved in the provision of health services.^5^ There are primary, secondary and tertiary levels in the country. According to the SNPP, there are:

…1,018 449 inhabitants in Swaziland with about 78.9% residing in rural areas. 52% of the population is under the age of 20 years. The capital is Mbabane (population 69 000) and the largest city is Manzini (population 75 000). The Government, private sector, non-governmental organizations (NGOs), faith based organisations and traditional healers are involved in the provision of health services. There are 8 government hospitals, 2 mission hospitals, 1 industry supported hospital, 8 public health units, 5 health centres, and 218 clinics (type A+ B). In addition there are 73 mission health facilities (health centres, clinics and outreach sites), 62 private clinics and 22 industry-supported health centres and clinics.^2, 5^

Swaziland has no established domestic pharmaceutical manufacturers. All medicines and medical supplies are imported. In the public sector, procurement is through an open tendering system. Procurement is restricted to items on the Swaziland National Essential Medicines List. Currently, there is no regulation for medicine pricing and thus no regulated dispensing fees. Medicine registration is not done in the country because the Pharmacy Act of 1929 does not have a process for registering and scheduling medicines, or licensing of sites for different types of pharmacies or inspections of such premises.^6^ A new Pharmacy Bill and a Medicines and Related Substances Control Bill have been developed which provides for the establishment of a Pharmacy Council and a Medicines Regulatory Authority.^7^

Medicines are free at the point of delivery in the public sector, with a nominal user fee of USD1.16 charged for consultation in hospitals, USD0.58 for radiology and USD0.35 for laboratory tests (1USD = SZL8.6570 according to the exchange rate as at 12 December, 2012, the Central Bank of Swaziland). These may stand as a barrier regarding access to services for those with low or no income capacity. The weekly income for the lowest-paid government worker in Swaziland is USD44.46.^8^

Chronic shortages of medicines in public health facilities have been observed,^5^ meaning that consumers are then forced to buy medicines in the private sector. Reliable evidence is required in order to understand what the specific problems are for one to come up with the best way to improve the situation, as well as whether governmental controls are necessary so as to ensure that essential medicines are available at the least cost to the consumer.

The aim of this study was to determine medicine prices, price structure, availability and affordability in the public health and private retail pharmacies in Swaziland. The objectives were to determine availability of medicines in both sectors, the differences between originator brand and generic medicine pricing, affordability of selected medicines and a comparison of prices in Swaziland against international reference prices. In addition, this study sought to identify the major cost drivers of medicine prices in Swaziland by examining the price components of selected medicines.

## Research methods and design

### Study design

A quantitative cross-sectional descriptive study design was employed.

### Setting

The study was conducted in one administrative area (Manzini region) of Swaziland. Manzini City, which is within the Manzini region, is the largest city in Swaziland, with a population of 75 000.^9^ The city is situated in the centre of the country and is considered to be the industrial capital, with various textile industries. There are three public hospitals and a public health unit located within the city. Manzini region has the largest number of licensed private retail pharmacies as compared with the other regions. More than 70% of the retail pharmacies in this region are located in Manzini City.

### Study population and sampling strategy

Both the public and private sectors were examined. Ten private sector outlets were selected at random from an updated list of 14 registered pharmacies within Manzini. The public sector sample of 10 outlets included two national referral hospitals and two regional hospitals. The remaining six outlets were selected at random from the list of clinics and public health units providing treatment and care for non-communicable diseases.^8^

A total of 16 chronic disease medicines were included in the survey, of which four form part of the World Health Organization – Health Action International (WHO–HAI) list of global medicines, one is regional and 11 are supplementary. The sample selection was based on the disease burden of the country and only medicines found in the essential medicines list were selected. According to the Health Management Information System report of 2009, diabetes was reportedly amongst the top 10 leading causes of inpatient admissions.^8^ Others that have added to the burden include cardiovascular diseases and cancers.^10^ The selected medicines all have an International Reference Price (IRP). The 2011 Management Sciences for Health (MSH) reference prices were used.^11^

### Data collection

Data collection took place between 12 December 2012 and 25 January 2013. The standardised methodology developed by WHO–HAI was used to conduct the survey.^1^ As medicines in the public sector are free to all,^8^ only data on medicine availability was collected from the public health facilities. Public-sector procurement prices were extracted from the procurement unit at the Ministry of Health. Private-sector patient prices and availability data were collected from the private retail pharmacies. To assess affordability, three common diseases namely asthma, hypertension and diabetes were selected and affordability of treatment was calculated using the daily wage of the lowest-paid unskilled government worker. A monthly treatment course was derived by multiplying the daily dose by 30. Price components data were collected in order to determine the various components that contributed to the final price of medicines.

Data were collected for two products, namely the originator brand (OB) and the lowest-priced generic equivalent (LPG) for each medicine found at each facility. Price data were not entered for products that were temporarily out of stock. Percentage availability was checked before leaving the facility to determine whether a back-up facility would have to be visited in order to conduct the survey again for those facilities with less than 50% of the survey medicines. To ensure data quality, data collection forms were reviewed every day after completion of the field work. Completeness of the forms, accuracy of unit prices and OB and LPG prices were double checked. Validation was undertaken for 20% of the facilities. These were selected randomly.

Five medicines were chosen for the price components study: Enalapril tablets, Atenolol tablets, Glibenclamide tablets, Metformin tablets and the Salbutamol inhaler. Key informants in the Ministry of Health, Ministry of Commerce and Trade and the Swaziland Revenue Authority were interviewed. Individual medicine prices were tracked from the time they are procured from manufacturers until they reach the patient. Information was obtained by interviewing both wholesalers and retailers.

### Data analysis

Data were entered into a pre-programmed Microsoft Excel workbook (Part I).^12^ Data entry was verified using the double entry and data-checker functions of the workbook. Summary tables and analyses such as median price ratios (MPRs) for all medicines, median MPRs, and percentage availability were generated automatically. The MPR is the ratio of the median price for each medicine across facilities divided by an IRP converted into local currency and is calculated when data are available from at least four facilities.

The availability of individual medicines was determined as being the percentage of facilities in which the medicine was found on the day of data collection. Mean availability was reported for the overall availability of the basket of medicines. Difference in percentage availability for OBs and LPGs of individual medicines and difference in average percentage availability for all OBs and LPGs would determine if the availability varies significantly between OBs and LPGs. Price components data were entered into a pre-programmed workbook (Part II).^13^

### Ethical considerations

The study and the tools received ethical clearance from the Humanities and Social Sciences Research Ethics Committee, University of KwaZulu-Natal (HSS/1188/012M). For access to the Manzini healthcare premises and data, permission and ethical clearance was granted by the Ministry of Health, Swaziland.

## Results

### Availability

Mean availability of LPG medicines in the private sector was 77.50% ± 27.7% compared with 68.00% ± 22.3% in the public sector. Mean availability of OB medicines was higher in the public sector (80%) versus the private sector (40%). Medicines that had a particularly low availability in the public sector include aminophylline (30%) and propranolol (40%). Amlodipine, captopril and glibenclamide were found in over 90% of the outlets visited in the public sector. In the private sector, the LPGs for aminophylline and irbesartan had very low availability (10% and 20% respectively), whilst atenolol, enalapril, glibenclamide, metformin and nifedipine were found in all the outlets.

### Public-sector procurement prices

Only one price data point was found for any of the OB products since the government purchased medicines by generic names. Based on the MPRs’ results for all 16 survey medicines, the public sector is procuring medicines at a slightly lower price than their IRPs (median MPR = 0.96). Half of the lowest-priced generic medicines were priced at 0.79 (25th percentile) to 1.72 (75th percentile) times their IRP. Medicines that were purchased at significantly less than IRPs include atenolol (0.65), captopril (0.72), irbesartan (0.25) and metformin (0.63). Examples of medicines for which the government was paying several times their IRPs include amlodipine (6.96), enalapril (3.86), phenytoin (7.56) and propranolol (2.97).

### Private-sector patient prices

When private-sector medicine prices were compared with IRPs, the OB products were found to be priced at 32.4 times the IRPs. Within the basket this varied from 3.56 for irbesartan to 196.51 for glibenclamide. The median price of the lowest-priced generic equivalent was 7.32 times the IRP price. However, within the basket this varied from 1.50 for a salbutamol inhaler to 61.89 for amlodipine. To facilitate comparison of prices between the two product types, only those medicines where both the OB and LPG were available were included in the analysis. The results indicate that in the private sector, OBs cost 473% more, on average, than their generic equivalents. The median MPR for OB products in the private sector was 41.06 (range of 4.25–168.75) whilst for LPGs it was 8.67 (range 1.68–53.66). MPRs for the medicines are summarised in Table 1.

**TABLE 1:** Median price ratios for selected medicines, originator brand and generic equivalents in the private sector.

Medicine	Originator	Generic
Amlodipine 10 mg cap/tab	104.74	53.66
Atenolol 50 mg cap/tab	100.53	8.67
Carbamazepine 200 mg cap/tab	23.83	6.07
Enalapril 20 mg cap/tab	20.62	12.31
Glibenclamide 5 mg cap/tab	168.75	11.02
Metformin 500 mg cap/tab	5.54	3.29
Nifedipine Retard 20 mg cap/tab	44.93	5.19
Phenytoin 100 mg cap/tab	41.06	8.69
Salbutamol inhaler 100 mcg/dose	4.25	1.68

mg, milligrams per dosage form; cap/tab, capsules or tablets as a dosage form; mcg/dose, micrograms per dose of medicine.

### Affordability of standard regimens

Affordability was measured only for medicines purchased from the private sector, since the government supplies medicines for free. The affordability of the LPGs, with the exception of enalapril, was less than one, with standard treatment costing slightly above a day's wage (1.2). Standard treatment with OBs cost more than a day's wage, except for metformin (0.9). Treating diabetes with glibenclamide required 6.7 days’ wages and treating hypertension with atenolol needed 4.8 days’ wages.

### Price components

Price components for imported products were measured to investigate the differences in mark-ups and to determine the impact of tariffs, taxes and mark-ups on the price the patient pays. The country has no price-control regulation for medicines. No maximum mark-ups are set for the wholesaler or retailer. Over-the-counter medicines are charged 14% value added tax (VAT). Public-sector procurement is by open tender method, but it is limited to the Essential Medicines List. The tender price is Delivery Duty Paid. The price of the medicine is fixed for the duration of the tender contract period. The public sector has a generic medicine policy which does not apply to the private sector.

The mark-ups identified from the survey displayed wide variations for different medicines within the sector (Table 2). Retail mark-up contribution to the final price was the highest mark-up observed and ranged from 31% to 53%, the lowest being enalapril for both the originator and generic product. Metformin had the largest retail mark-up contribution to final price, with wholesale mark-up contribution being between 20% and 29%. The Cost, Insurance and Freight (CIF) contributed 18% to 34%. The landed price component (that is the price of the medicine after it arrives in a country and has passed all custom and import requirements and is transported to its first destination in the country) contributed from 4% to 8% of the final price.

**TABLE 2:** Percent contribution of price components to final medicine price, originator vs. generic brands for selected medicines.

Medicine	Cost, insurance and freight	Landed price	Wholesale	Retail/dispensed price	Total percentage
Metformin 500 mg (OB)	18	4	27	51	100
Metformin 500 mg (LPG)	22	5	20	53	100
Salbutamol 100 mcg/dose (OB)	27	6	22	45	100
Salbutamol 100 mcg/dose (LPG)	34	8	26	32	100
Atenolol 50 mg (OB)	26	5	22	47	100
Atenolol 50 mg (LPG)	25	5	22	48	100

OB, originator brand; LPG, lowest-priced generic equivalent; mcg/dose, microgram per dose of medication.

The total cumulative mark-up was greater than 200% for all of the medicines with the exception of a generic salbutamol inhaler which had the lowest cumulative mark-up (190.99%). Metformin OB had the highest cumulative mark-up (440.27%), whilst glibenclamide and atenolol LPGs had a higher cumulative mark-up than the OBs (430.04% and 305.63% respectively). However, the patient still paid much less when buying these generics because their CIF price was low. Price components for enalapril were similar for the generic and originator products. It was also observed that the CIF price for the generic type was 94.8% that of the originator.

## Discussion

In the public sector, only one of the survey medicines was found in the form of the OB product. This shows effective generic policy implementation in the public sector. In the private pharmacies, both the originator brands and generic equivalents were found, although generics were more widely available. Procurement prices in the public sector were very competitive, with medians below the IRPs. However, certain medicines such as phenytoin (MPR = 7.55) and amlodipine (MPR = 6.96) were priced considerably higher than their IRPs. The final patient prices for both LPGs and OBs were high in the private sector in terms of international pricing. LPGs were priced at nearly eight times their IRP, whilst OB medicines were priced at nearly 33 times their IRP. These disparities suggest substantial variation in price mark-ups between medicines.

The affordability of LPGs was generally good for all medicines, with standard treatment costing about a day's wage or less than the daily wage of the lowest-paid government worker in the private sector. However, the lowest-paid government worker is paid much more than the majority of the population. All the survey medicines were exempt from VAT as they are prescription medicines. The largest contributor to add-on costs was the retail mark-up. Generally, high mark-ups at different points in the supply chain can drastically increase prices and make medicines less affordable. Therefore, prices of medicines can be lowered substantially by reducing or regulating the mark-ups. Generics are more affordable than OBs and they would be more so if mark-ups were regulated.

About 63% of the population in Swaziland is living below the upper poverty line of USD8.21 per capita per month and, therefore, treatments which have been calculated to be affordable may still be too costly.^3^ In addition, these costs do not include the other costs such as consultation and diagnostic tests. Finally, families generally require medicines for more than one member and may be confronted with unaffordable drug costs. Patients who cannot afford the private-sector costs may do without medicines completely. These findings are in line with other studies of affordability of chronic medicines which indicate that these medicines are unaffordable for many populations.^14, 15, 16, 17^

### Strengths and limitations

A major strength of this study is the use of the WHO–HAI medicine prices. The survey has allowed for the measurement of medicine prices and affordability in a reliable and standardised way that enables valid international comparisons to be made.

A limitation of the study is that availability is determined only for the list of survey medicines and therefore does not account for the availability of alternate forms, or of therapeutic alternatives for these medicines. Another limitation is that the MSH reference price is a median of recent procurement prices offered by for-profit and non-profit suppliers to international non-profit agencies for mostly generic products. This means that the MSH reference price is dependent on the number of supplier prices used which also determines the reliability of the MPRs. In cases where very few or no supplier prices are available and the buyer price is used as a proxy, MPR results can be skewed by a particularly high or low IRP. The quality of the products surveyed is another limitation of the study. The medicines are not yet registered in Swaziland and quality control testing was not performed. Assumption is made that they were of acceptable quality.

### Recommendations

The findings of this study support the objectives of the SNPP, namely, to implement pricing policies to make medicines more affordable and available and to encourage generic prescription and dispensing as well as generic substitution. There is a need to uphold the generic policy implementation in the procurement of medicines in the public sector. Awareness creation and promotion of generic acceptance in the community and amongst healthcare professionals should be done. The use of IRPs as a benchmark should be encouraged in order to ensure lower procurement prices in the public sector.

A more comprehensive WHO–HAI based medicines price survey should be carried out to include more sectors and other essential medicines. There is clearly a need for a pricing policy to improve the availability of affordable generics. It should contain aspects of price control and incentives in order to reduce prices.

## Conclusions

About one-third of the global population lacks reliable access to needed medicines, with this proportion being as high as 50% in some of the poorest countries of Africa and Asia.^18^ One of the factors contributing to this lack of access is the price of medicines in countries. Whereas high-income industrialised countries often have health insurance systems, so that out-of-pocket payments for medicines are only required by 20% of the population, a lack of social insurance systems in developing nations contributes to the fact that up to 90% of people buy medicines through out-of-pocket payment.^19^ The study has provided some insight into current issues related to price, availability and affordability of key medicines for the treatment of common chronic disease conditions in Swaziland. It has supported the need for policy development in terms of pricing policies and regulation of mark-ups that are required to increase availability, reduce prices, and improve affordability, which is the goal of the SNPP.
